# Knockdown of ENTPD5 inhibits tumor metastasis and growth via regulating the GRP78/p-eIF-2α/CHOP pathway in serous ovarian cancer

**DOI:** 10.1186/s13048-022-00996-0

**Published:** 2022-06-07

**Authors:** Xueping Chen, Zhiqiang Zha, Yu Wang, Yun Chen, Menglan Pang, Liping Huang, Yao Chen

**Affiliations:** 1 Department of Obstetrics and Gynecology, Nanfang Hospital, Southern Medical University, Guangzhou, 510515 Guangdong China; 2 School of Medical Laboratory and Biotechnology, Southern Medical University, Guangzhou, 510515 Guangdong China

**Keywords:** ENTPD5, Serous ovarian cancer, Proliferation, Migration, Endoplasmic reticulum stress, Unfolded protein response

## Abstract

**Background:**

Dysregulation of Ectonucleoside Triphospahate Diphosphohydrolase 5 (ENTPD5) in tumors might be associated with tumor progression, while the role of ENTPD5 in the growth and metastasis of serous ovarian cancer (SOC) is still unclear.

**Methods:**

ENTPD5 expression patterns in ovarian cancer tissues were analyzed by qRT-PCR and immunohistochemistry assay (IHC). Two SOC cell lines, SKOV3 and OVCAR8, were stably transfected with lentivirus to build knockdown and overexpression cell lines. Clone formation assay, collagen gel droplet culture technology, wound healing assay and flow cytometry were used to assess the migration and growth traits of SOC cells. Expression levels of ENTPD5, glucose regulated protein 78 (GRP78), eukaryotic translation initiation factor 2 alpha (eIF-2α), phosphorylated -eIF-2α and, C/EBP homologous protein (CHOP) in SOC cells were detected by Western blot.

**Results:**

Compared to fallopian tube tissues, the expression of ENTPD5 was significantly higher in tumor tissues obtained from SOC patients, and positively correlated with clinical stage and metastasis. ENTPD5 knockdown robustly inhibited cell proliferation, migration, whereas ENTPD5 overexpression elicited the opposite effect on SOC cells. ENTPD5 knockdown arrested cell cycle in G0/G1 phase and increased apoptosis. Importantly, ENTPD5 knockdown was associated with significantly decreased protein levels for GRP78, CHOP, and p-eIF-2α, suggesting possible involvement of ENTPD5 in endoplasmic reticulum stress (ERS).

**Conclusions:**

Our study demonstrates that ENTPD5 knockdown inhibited SOC cell proliferation, migration and restrained the activation of the GRP78/p-eIF-2α/CHOP pathway, which provides a potentially effective therapeutic target for the treatment of SOC.

**Supplementary Information:**

The online version contains supplementary material available at 10.1186/s13048-022-00996-0.

## Introduction

Ovarian cancer is one of the most malignant tumors that seriously threaten women's health [1]. The vast majority of ovarian tumors are epithelial ovarian cancer, which accounts for 85–90% of ovarian malignancies [2]. Epithelial ovarian cancer can be subdivided into four major types: serous, endometrioid, mucinous, and clear-cell carcinomas. Moreover, SOC accounts for a large majority (~ 70%) of epithelial ovarian cancer [3, 4]. Due to its onset being hidden and symptoms appearing late, the fatality rate caused by ovarian cancer ranks first among gynecological malignancies [5–7]. More than 80% of newly diagnosed patients are in advanced stages, and tumor metastasis occurs in more than 70% of these cases [8]. Therefore, ovarian cancer is known as the "hidden killer" for women's health [5, 7]. Therefore, there is an urgent need to understand the pathogenesis of ovarian cancer to establish new treatment and diagnostic strategies for this deadly disease.

ENTPD5 is a nucleotide hydrolase, mainly present in the endoplasmic reticulum and mediates the catabolism of nucleotides in cells. ENTPD5 can hydrolyze UDP to UMP, thereby promoting the glycosylation and glycoprotein folding of proteins, especially the tyrosine protein kinase receptor [9–13]. Recent studies have shown that the mutated tumor suppressor gene PTEN can regulate the expression of ENTPD5 [14]. The overexpression of ENTPD5 in melanoma cells could enhance the survival of tumor cells, resist endoplasmic reticulum stress-mediated apoptosis, and promote tumor cell metastasis [15, 16]. The latest PNAS research reports showed that ENTPD5 is a target molecule of the mutant tumor suppressor gene p53, regulating the development and metastasis of pancreatic cancer [17]. The study by Read et al. strongly suggests that deletion of ENTPD5 promotes hepatoma formation in mice [18]. However, the function and molecular mechanism of ENTPD5 in ovarian cancer have not yet been elucidated. Therefore, this study explored the role of ENTPD5 in ovarian cancer cells and its underlying mechanisms.

## Materials and methods

### Patients and tissue samples

ovarian tumor specimens were obtained from a cohort of patients treated at Nanfang Hospital, affiliated with Southern Medical University, China, between 2013 and 2020. The OC specimens were from primary ovarian cancer patients with no previous surgery or chemotherapy. The normal FT tissues were derived from patients who had a benign gynecologic tumor and received hysterectomy and bilateral salpingo-oophorectomy. The distribution of tumor characteristics for SOC patients was as Table S1. This study was approved by the Research Ethics Committee of Southern Medical University (approval number: NFEC-2021–260). And all the patients were informed and agreed to participate in this study. Investigations involving humans will have been performed in accordance with the principles of Declaration of Helsinki.

### Cell lines and cell culture

Human serous ovarian cancer cell lines SKOV3, OVCAR8 were purchased from the cell bank of the Chinese Academy of Sciences (Shanghai, China). SKOV3 and OVCAR8 were cultured in DMEM culture solution (Life Technologies, Carlsbad, CA, USA) containing 10% fetal bovine serum (Life Technologies, Carlsbad, CA, USA), respectively, and placed in an incubator at 5% CO2, 37℃and saturated humidity.

### Establishment of stable cells

The lentiviruses for ENTPD5 overexpression, ENTPD5 shRNA and empty vector were purchased from Genechem (Shanghai, China). SKOV3 and OCAVAR8 cells in logarithmic growth phase were plated in 6-well plates with a planting density of 1 × 10^5^ / well, and cultured in a 37 °C incubator. According to the instructions, when the cell growth density reached 30%, the infection was performed for 24 h. The puromycin medium was changed to screen positive cells at 24 h. The cells infected with ENTPD5 shRNA and overexpressed lentiviruses were recorded as sh-ENTPD5 group and LV-ENTPD5 group, and the cells infected with negative control were recorded as NC group. The sequences of short hairpin RNA (shRNA) targeting ENTPD5 were: 5′-CCAACACCATGCGTGTTGT-3′; The invalid shRNA sequences were: 5′-TTCTCCGAACGTGTCACGT-3′.

### qRT⁃PCR analysis

qRT-PCR analysis was performed using standard procedures according to SYBR premix Ex Taq Kit detection kit (Applied Biosystems). The internal reference gene was 18SRNA, and the qRT⁃PCR reaction program was set to 2 steps:(a) pre-incubation at 95 °C for the 30 s; (b) 40 PCR cycles of 95 °C for 5 s, 55 °C for 30 s, 72 °C for 34 s. Samples were assayed in triplicate using the ABI Prism 7500 detection system (Perkin Elmer Applied Biosystems). The relative quantization value was then calculated by subtracting the average CT from the corresponding average CT for 18S rRNA. Primer sequences were as Table S2.

### Western blot analysis

The protein was extracted with RIPA lysis buffer and quantified using the Bicinchoninic Acid (BCA) Protein Assay Kit (Beyotime, Shanghai, China). The sample was resolved using SDS-PAGE and then transferred onto PVDF membranes (Millipore, MA, USA). Nonspecific binding was blocked by 5% nonfat milk for 2 h at RT, and the membrane was then washed with Tris-buffered saline-Tween 20. The membranes were incubated with primary antibodies targeting GRP78, eIF-2α, Phosphorylated-eIF-2α, CHOP (1:1000, Beyotime, Shanghai, China), ENTPD5 and GAPDH (1:1000, Abcam, USA) overnight at 4 °C.Then, the membrane was incubated with secondary antibody for 2 h at RT. Then immunoreactive proteins were visualized with the ECL detection system (Millipore, MA, USA).

### Clone formation assay

200 cells were planted in each well of 6-well plastic plates. Each type of cell was planted in 3 wells. The cells were cultured at 37ºC, with 5% CO_2,_ and under saturation humidity for 21 days. Cells clones with more than 50 cells were counted under an inverted microscope. Clone form rate was calculated as follows: (Average clone number of 3 wells)/(number of plating cells)*100%. Cells clones were fixed with paraformaldehyde and stained with crystal violet.

### Wound-healing assay

Cells were seeded on a 6-well plate and were grown to get 90% confluence. Wounds were created by using 200 µl pipette tips to scratch a straight line. The wound areas were imaged at 0 h, 24 h under a microscope. Image J software (NIH, USA) was used to calculate the wound areas.

### Flow cytometry for Apoptosis and Cell cycle analysis

Cells (1 × 10^4^ /mL) were seeded in 24-well plates and starved for 24 h to uniform the cell cycle. The cells are trypsinized and collected after replacing the fresh medium for 24 h and cell cycle assay was performed with 50 µg/mL propidium iodide (PI, Sigma, Shanghai, China). Cells (1 × 10^4^ /mL) were seeded in 24-well plates for 24 h and cell apoptosis assay was performed by FITC-Annexin V Apoptosis Detection Kit (Biolegend, San Diego, CA, USA) according to the manufacturer's instructions. All cells were acquired by FACS Calibur flow cytometer (BD Pharmingen, San Diego, CA, USA), and the results were anglicized with Flowjo software and collecting 10,000 events for each sample.

### Immunohistochemistry

Ovarian cancer specimens are surgical resection specimens, and the specimens are fixed with 10% neutral formaldehyde after removal from the body. Immunohistochemistry was done using 8 μm serial sections placed onto glass slides using a single-staining procedure. The protocols used with each antibody are carried out according to the instructions of the Immunohistochemistry kit. Comprehensive analysis and scoring were performed according to the respective staining degree and number of stained cells. The intensity was scored as negative (0), weak (1), medium (2), and strong (3). and the proportion of staining was scored as 1 (≤ 10%), 2 (11–50%), 3 (51–75%), and 4 (> 75%). An overall expression score was calculated by multiplying the scores for intensity and proportion, ranging from 0 to 12 [19].

### 3D growth model build by collagen gel droplet culture technology

Briefly, type I collagen (Cellmatrix Type CD; Nitta Gelatin, Inc., Osaka, Japan), 10X F-12 medium, and reconstitution buffer were mixed together at a ratio of 8:1:1. The prepared tumor cells and collagen solution are inoculated at a volume ratio of 10:1, so that the final density of cells in the collagen droplets is 2 × 10 ^5^ ~ 5 × 10 ^5^ pieces/mL. The collagen-cell mixture (30 μl/drop) was transferred to the 6-well multiplate and cultured at 37 °C in a CO_2_ incubator. After culture for 13 days, each collagen droplet was stained with neutral red, fixed with 10% neutral formalin buffer, washed with water, and quantified by culture cell analysis system. Image analysis at 540 nm would quantify the number of ENTPD5 knockdown cells and control cells.

### Statistical analysis

Graphpad prism 5.0 was used to generate the graphs and process the data. Data were expressed as the mean ± SD. Comparison of two groups was performed with Student’s t test with a two-tailed *p* value. Chi-squared test was used to analyze the differences in clinical characteristics. *p* < 0.05 were considered significant.

## Results

### ENTPD5 is overexpressed in human SOC tissues and was associated with tumor malignancy

**Fig. 1:**
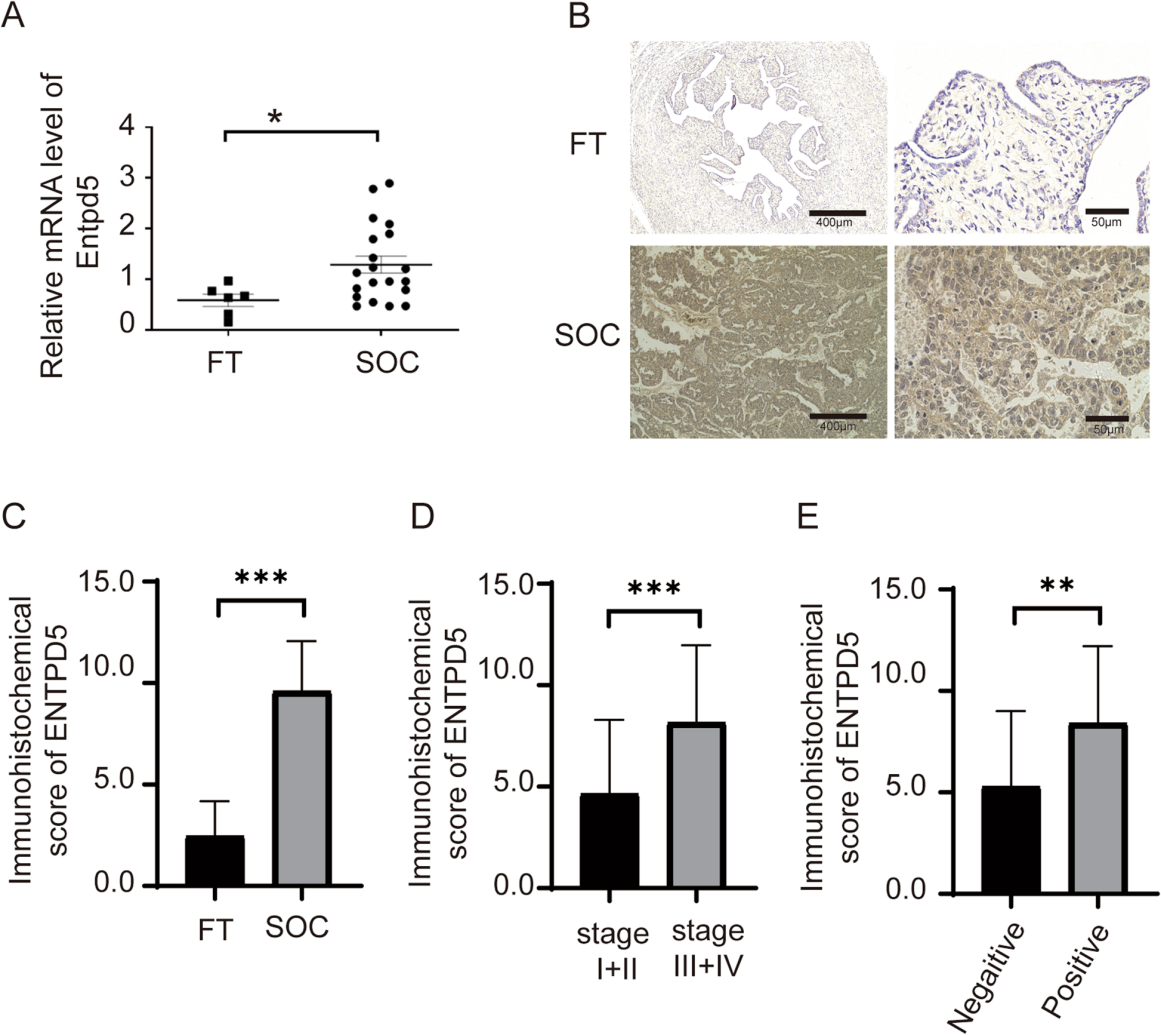
ENTPD5 is overexpressed in human OC tissues and was associated with tumor metastasis. **A** qRT-PCR analysis of the mRNA level of ENTPD5 in serous ovarian cancer (SOC) tissues and normal fallopian tube tissues (FT) (FT, *n* = 6; SOC, *n* = 20). **B** Representative images of IHC staining of ENTPD5 in SOC tissues and FT tissues (left, × 100; right, × 600). **C** Immunohistochemical score of (B). Immunohistochemical score according to FIGO stage **D** and omentum metastasis **E** of SOC. (data are mean ± SD, *p < 0.05, ***p < 0.001)

In order to clarify the molecular mechanism of ENTPD5 in ovarian cancer(OC), we first evaluated the ENTPD5 expression levels in OC and normal fallopian tube tissues (FT), the results from qRT-PCR showed that the mRNA levels of ENTPD5 were significantly higher in OC tissues than in FT tissues (Fig. 1A, *p* < 0.05). Furthermore, 6 FT and 82 OC specimens were analyzed by immunohistochemical staining. The expression of ENTPD5 was weak or almost no staining in FT tissues, and a strong positive signal of ENTPD5 was detected in the cytoplasm of OC tissues (Fig. 1B). The vast majority of the patients (79.27%, 65/82, Table S1) were serous ovarian cancer. Therefore, we analyzed the expression of ENTPD5 in SOC and FT tissues. As shown in Fig. 1C, the immunohistochemical score of SOC tissues was higher than that of FT tissues (*p* < 0.001). Next, we analyzed the relationship between ENTPD5 and the clinical characteristics of SOC. SOC specimens were divided into the low ENTPD5 expression group and high ENTPD5 expression group based on IHC scores. Clinicopathological characteristic analysis showed that ENTPD5 expression was positively correlated with the clinical FIGO stage (Fig. 1D, *p* < 0.001) and omentum metastasis (Fig. 1E, *p *< 0.01), but no significant correlation with age, CA125 level and lymph node metastasis (Table 1). All these results showed that ENTPD5 may be closely related to the malignancy of SOC.

**Table 1:** Correlation between ENTPD5 expression and clinicopathological features of serous ovarian cancer patients

Clinicopathological features		ENTPD5 Expression level		
		Low	High	*P* Value
Ages(year)	< 56	13	20	0.18
	≥ 56	7	25	
FIGO stage	I + II	9	5	< 0.001***
	III + IV	7	44	
CA125(U/ml)	< 600	11	27	0.71
	≥ 600	9	18	
Lymph node metastasis	Positive	8	11	0.2
	Negative	12	34	
Omentum metastasis	Positive	9	35	< 0.01*
	Negative	11	10	

*p < 0.01, ***p < 0.001

### ENTPD5 knockdown inhibits proliferation of SOC cells

**Fig. 2:**
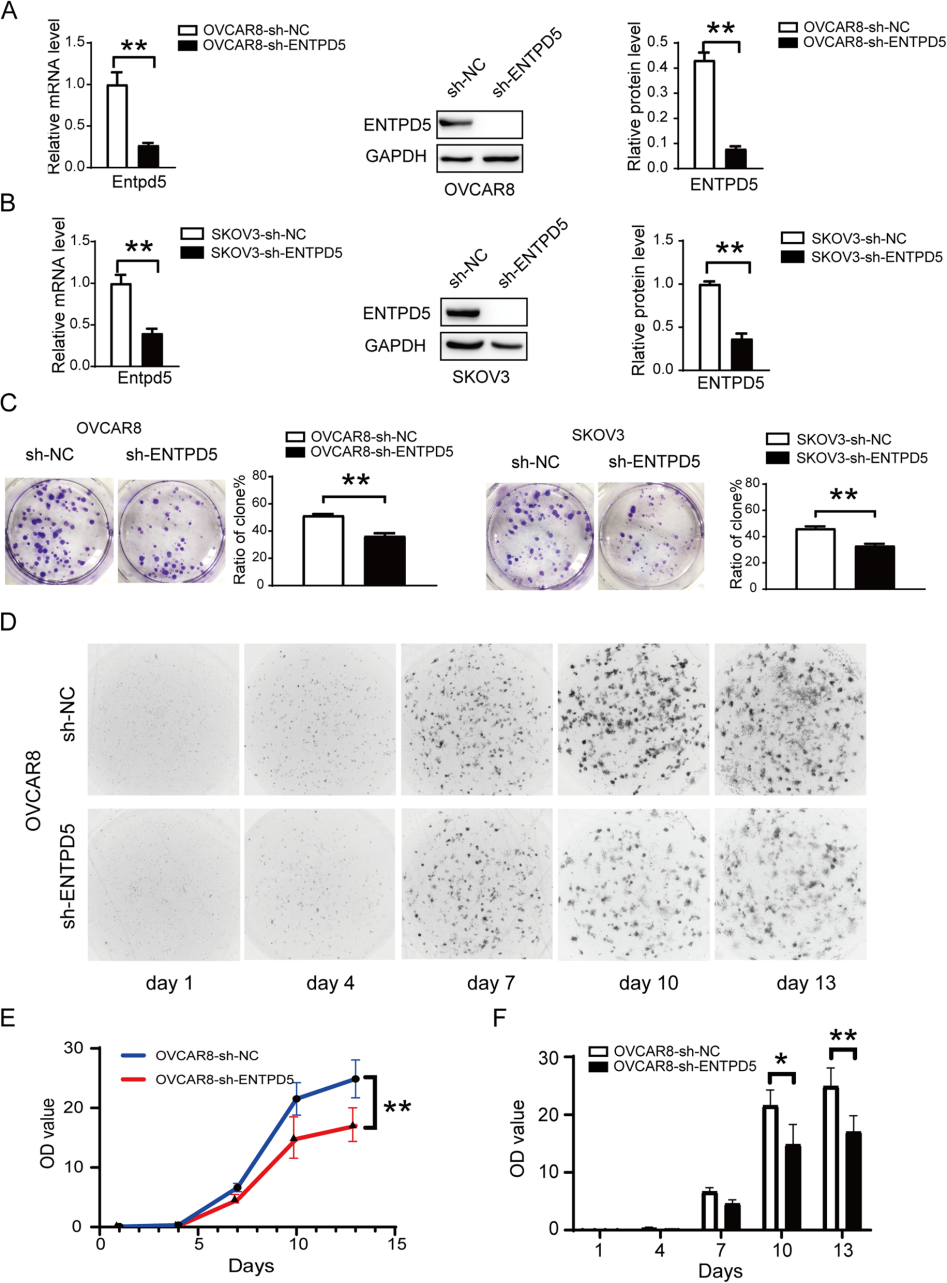
ENTPD5 knockdown inhibits cell growth of SOC cells in vitro. **A**, **B** OVCAR8 and SKOV3 cells were stably transfected with negative control (sh-NC), ENTPD5 shRNA (sh-ENTPD5), mRNA and protein levels of ENTPD5 and GAPDH were analyzed by qRT-PCR and western blot in OVCAR8 and SKOV3 cells. **C** Soft agar colony formation assays the colony forming ability of OVCAR8 and SKOV3 cells with ENTPD5 knockdown. **D** 3D cultured cells photo of OVCAR8 cell. **E** The 13-day growth curve of ENTPD5 knockdown in OVCAR8 cell. **F** The OD value of ENTPD5 knockdown in OVCAR8 cell. (data are mean ± SD, *p < 0.05, **p < 0.05)

Short hairpin RNA (shRNA) targeting ENTPD5 was clone into lentivirus vector to construct ENTPD5 knockdown cells, invalid shRNA sequences were also transfected as a negative control. ENTPD5 knockdown was confirmed by qRT-PCR and Western blot (Fig. 2A, B, *p* < 0.01). The results of soft agar colony formation assay indicated that ENTPD5 knockdown significantly reduced the colony forming ability compared to negative control cells (Fig. 2C, *p* < 0.01).

The 3D growth model of tumor cells in vitro can visually observe the growth and proliferation of tumor cells and simulate the growth state of cells in 3D culture model. The results of the 3D growth model showed that ENTPD5 knockdown resulted in a significant delay in tumor growth (Fig. 2D, E, *p* < 0.01). Cell quantification results showed that the difference in tumor cell count of ENTPD5 knockdown cells was statistically significant compared to the negative control after 13 days of culture (Fig. 2F, *p* < 0.01). These results support that the knockdown of ENTPD5 inhibits cell proliferation of ovarian cancer cells.

### ENTPD5 knockdown induces cell cycle arrest and promotes cell apoptosis

**Fig. 3:**
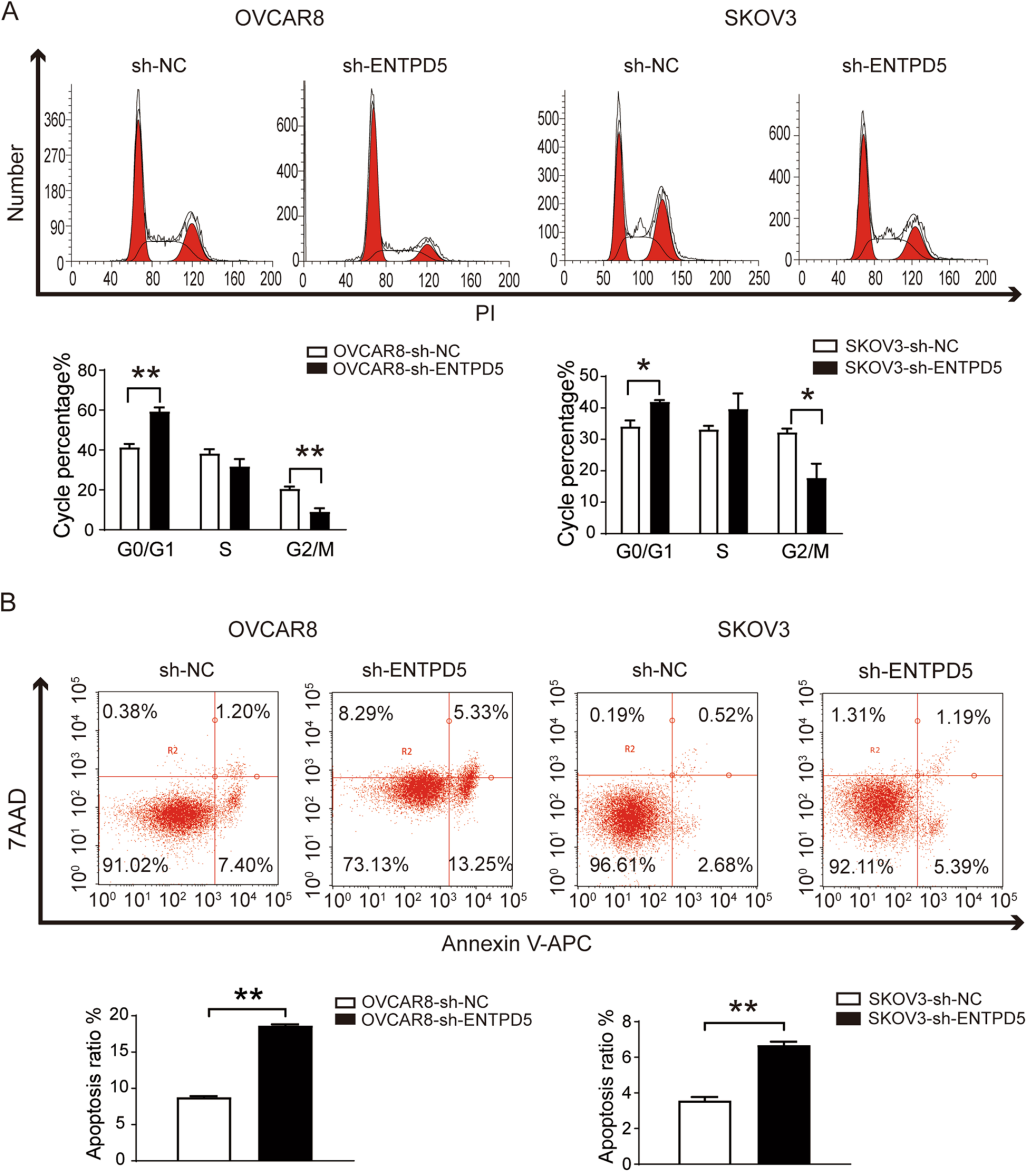
ENTPD5 knockdown induces G0/G1 cell cycle arrest and promotes cell apoptosis. **A**, **B** The proportion of apoptotic and cell cycle in OVCAR8 and SKOV3 cells were analysed by flow cytometry. (data are mean ± SD, *p < 0.05, **p < 0.01)

As shown in Fig. 3A, the results of Flow cytometry showed that ENTPD5 knockdown increased the proportion of cells in the G0/G1 phase (*p *< 0.01). Correspondingly, ENTPD5 knockdown decreases the proportion of cells in G2 phase both in OVCAR8 and SKOV3 cells(*p *< 0.05). Furthermore, the apoptosis ratio was increased significantly by ENTPD5 knockdown compared to the negative control in vitro (Fig. 3B,* p* < 0.01). The results showed that ENTPD5 knockdown induced G0/G1 phase arrest and promotes cell apoptosis.

### ENTPD5 knockdown attenuates migration capacity of SOC cells

**Fig. 4:**
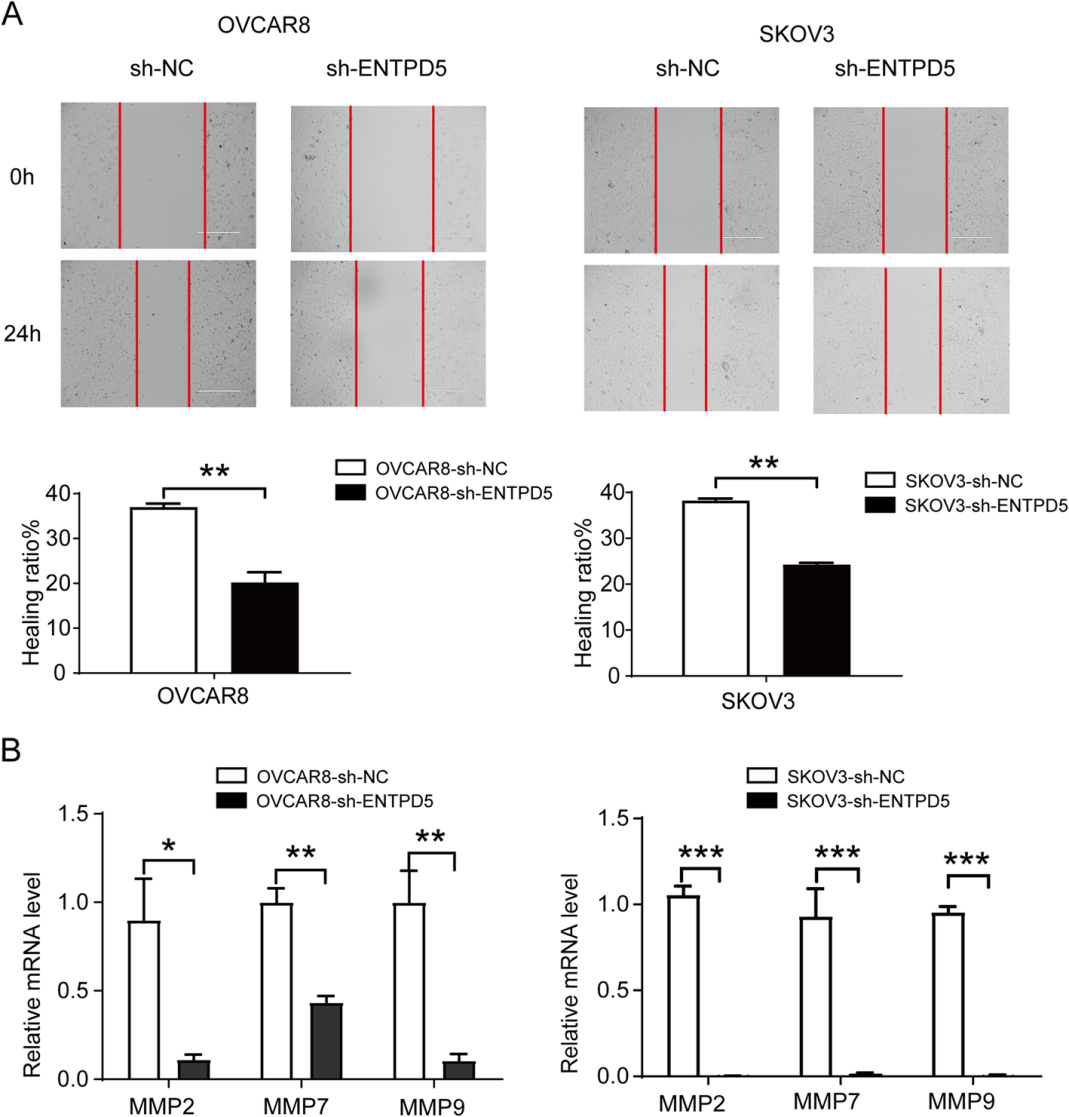
ENTPD5 knockdown attenuates migration capacity of SOC cells. **A **Wound-healing assays was performed to determinate the effects of ENTPD5 knockdown on migration of OVCAR8 and SKOV3 cells. **B** qRT-PCR was used to analysis the mRNA level of MMP2, MMP7, MMP9 of OVCAR8 and SKOV3 cells with ENTPD5 knockdown. (data are mean ± SD, *p < 0.05, **p < 0.01, ***p < 0.001)

Wound-healing assay suggested that ENTPD5 knockdown was associated with decreased migration abilities of both SKOV3 and OVCAR8 cells (Fig. 4A, *p* < 0.01). These results indicated that the overexpression of ENTPD5 in SOC might heighten the metastasis potential of tumor, which was accords with clinicopathological characteristic analysis. Matrix metalloproteinase (MMP) degrade structural proteins of invaded tissues and plays a crucial role in metastasis of tumor cells. The results of qRT-PCR showed that the transcription of MMP2, MMP7, MMP9 were downregulated by ENTPD5 knockdown both in SKOV3 and OVCAR8 cells (Fig. 4B, *p* < 0.05). These results support that ENTPD5 knockdown attenuates the migration capacity of SOC cells in vitro.

### ENTPD5 overexpression promotes SOC cell proliferation and migration in vitro

**Fig. 5:**
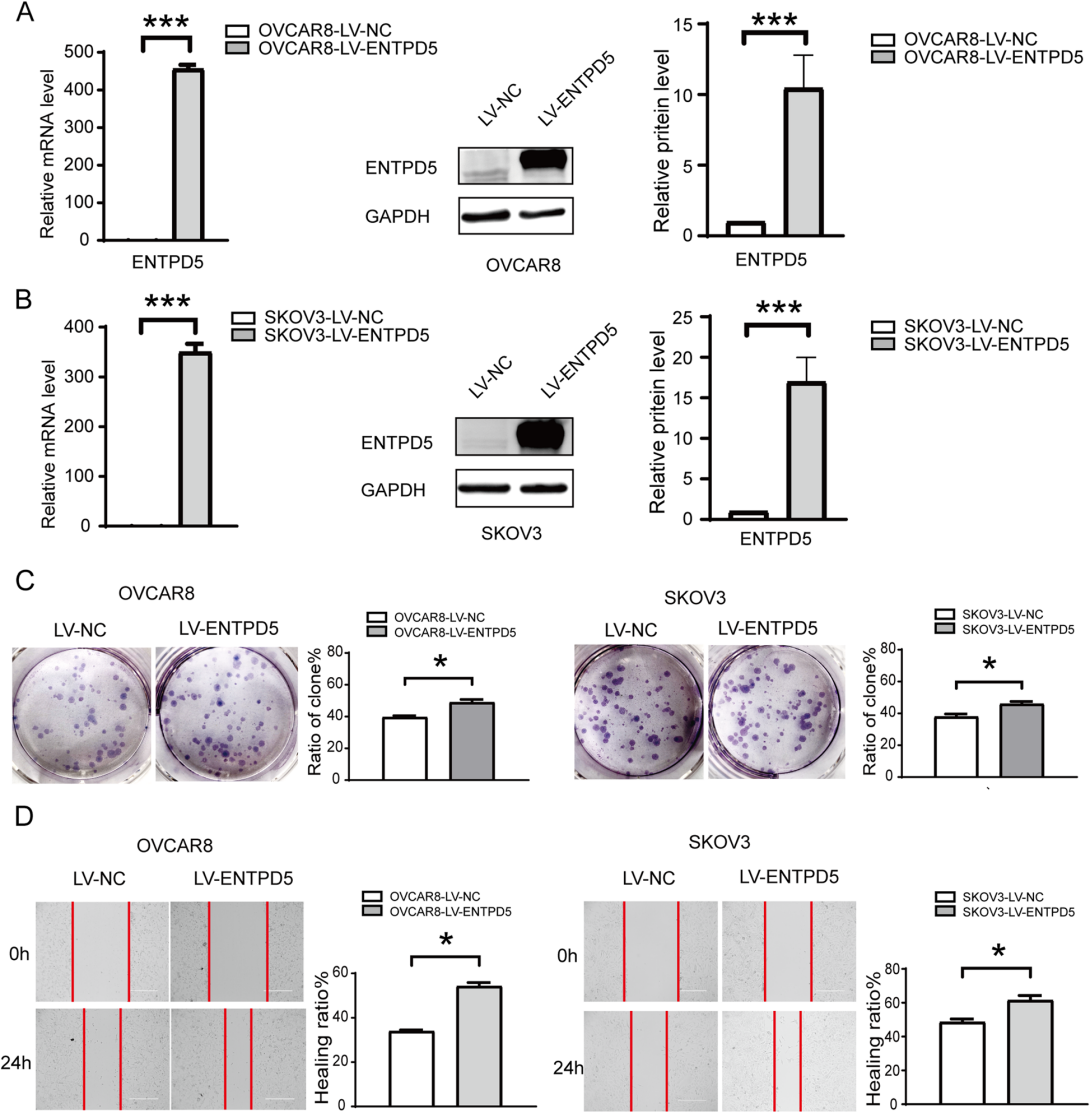
Overexpression of ENTPD5 can promote SOC cell proliferation and migration. **A**, **B** OVCAR8 and SKOV3 cells were stably transfected with negative control (LV-NC) and ENTPD5 overexpression (LV-ENTPD5), mRNA and protein levels of ENTPD5 and GAPDH were analyzed by western blot in OVCAR8 and SKOV3 cells. **C **Soft agar colony formation assays the colony forming ability of OVCAR8 and SKOV3 cells with ENTPD5 overexpression (LV-ENTPD5). **D** Wound-healing assay was performed to determine the effects of ENTPD5 overexpression on migration of OVCAR8 and SKOV3 cells. (data are mean ± SD, *p < 0.05, ***p < 0.001)

In order to further determine the role of ENTPD5 on the progression of SOC, ENTPD5 overexpression cells were built with SKOV3 and OVCAR8 cells. The results of qRT-PCR and Western blot showed that the ENTPD5 overexpression cells were constructed successfully (Fig. 5A, B, *p* < 0.01). The clone formation assay showed that ENTPD5 overexpression promoted the clone formation capacity in both SKOV3 and OVCAR8 cells (Fig. 5C, *p* < 0.05). Strikingly, wound-healing assay showed that ENTPD5 overexpression promoted SOC cell migration (Fig. 5D, *p* < 0.05). Combined with the results of ENTPD5 knockdown, ENTPD5 overexpression promoted the malignant characteristics of SOC.

## ENTPD5 knockdown suppresses the GRP78/p-eIF-2α/CHOP pathway

**Fig. 6:**
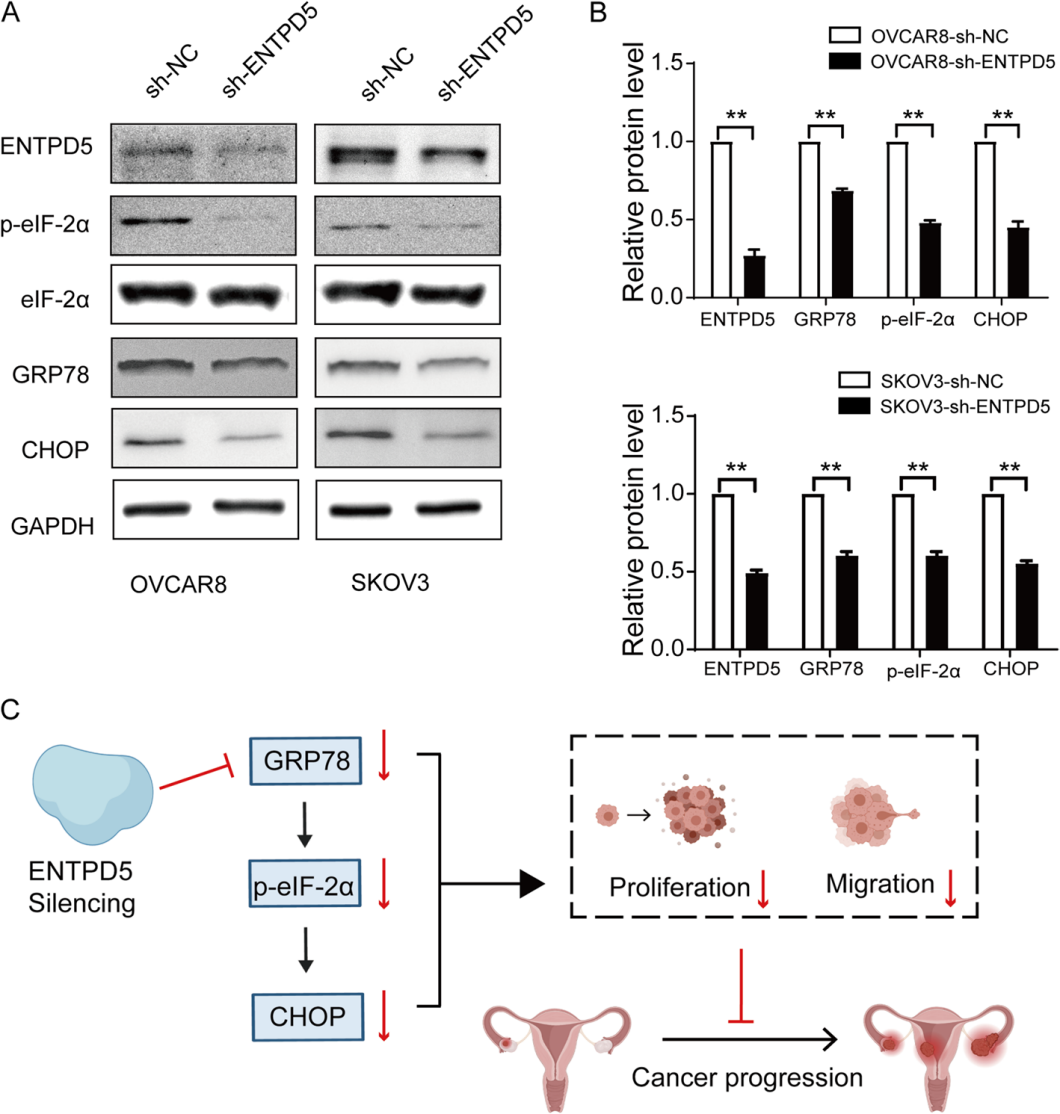
ENTPD5 knockdown suppresses the GRP78/p-eIF-2α/CHOP pathway. **A** Western blotting for basal expression of ENTPD5, GRP78, eIF-2α, p-eIF-2α and CHOP in SKOV3 and OVACAR8 ovarian cancer cell lines. GAPDH was used as a loading control. **B** Statistical analysis of (**A**). **C **Schematic plot summarizing regulation of ENTPD5 in ovarian cancer. (data are mean ± SD, **p < 0.01)

As shown in Fig. 6, expression level of GRP78, the central regulator of endoplasmic reticulum stress, was notably decreased after transfection of the short hairpin RNA (shRNA) against ENTPD5 in comparison with the control group (Fig. 6A, B, *p* < 0.01). The downstream signaling molecules of GRP78, phosphorylated eIF-2a and CHOP were also significantly down-regulated with ENTPD5 knockdown (Fig. 6A, B, *p* < 0.01). All these results indicated that ENTPD5 knockdown might suppress the GRP78/p-eIF-2α/CHOP pathway.

## Discussion

Ovarian cancer is the most lethal gynecologic malignancy. Currently, the clinical treatment for ovarian cancer is surgery combined with chemotherapy drugs [20]. Tumor recurrence and metastasis are major challenges in the treatment of ovarian cancer. In the present study, we characterized a novel ectonucleoside triphosphate diphosphohydrolase in human ovarian cancer known as ENTPD5 whose biological function was poorly declared [11, 12, 14, 21]. We have demonstrated that ENTPD5 was highly expressed in SOC tissues and positively associated with tumor metastasis. These results suggest that ENTPD5 may participate in SOC metastasis.

Previous studies have shown that the ENTPD5 is closely associated with tumor progression [14, 16]. Therefore, we investigated the effect of ENTPD5 in SOC cells. As shown by both the cell clone formation assay and FACS analysis, ENTPD5 knockdown resulted in a significant delay in tumor growth in vitro, and the cell cycle was arrested in the G0/G1 phase accompanied by a significant increase of apoptosis. In addition, we also observed ENTPD5 knockdown inhibited cell migration. By contrast, ENTPD5 overexpression significantly promoted cell proliferation and migration. MMP was considered to play an important role in predicting ovarian cancer outcome, especially related to cancer invasion, metastasis, and poor prognosis [22–24]. For the first time, we found that silencing ENTPD5 decreased the expression of metal matrix protease. 3D culture model can replicate the biological tumor microenvironment through cell-to-cell communication, and more truly reflect the biological functions of cancer cells [25, 26]. These results can explain that ENTPD5 knockdown reduces the growth of SOC cells in 3D culture based on collagen droplets.

Endoplasmic reticulum stress usually leads to the accumulation of unfolded or misfolded proteins in endoplasmic reticulum, causing unfolded protein response (UPR), triggering adaptive survival response or cell death [27]. UPR has become one of the possible targets for tumor therapy [28]. ENTPD5, a nucleotide hydrolase mainly existing in the endoplasmic reticulum, assists in protein folding and induces the degradation of misfolded proteins [29, 30]. In our study, we explored whether ENTPD5 could affect the biological behavior of SOC by regulating UPR.

GRP78, the central regulatory molecule of UPR, can activate UPR to reduce or stop ERS, so as to stabilize the homeostasis of cancer cells and exert an important role in UPR regulation, cell survival, proliferation and migration [31–33]. The dissociation of PERK and GRP78 can trigger the phosphorylation of eIF-2α, which reduces the overall translation frequency of cellular mRNA and induces the transcription of CHOP. As a member of the bZIP transcription factor protein family, CHOP can regulate a series of apoptosis-related factors and be involved in regulating the biological behaviors of tumor cells [15, 34]. As shown in this study, the expression level of ENTPD5 is proportional to activation of GRP78/p-eIF-2α/CHOP pathway. We speculated that high expression of ENTPD5 promoted in SOC may promote the degradation of unfolded protein and reduce the pressure of endoplasmic reticulum. These results may explain the reason why ENTPD5 knockdown reduces the proliferation and migration of SOC cells given the importance of ERS in tumorigenesis and tumor development. Altogether, our results suggest that ENTPD5 is important in the pathogenesis of SOC. Thus, targeting ENTPD5 may be a promising therapeutic strategy for SOC.

## Conclusion

In conclusion, our study demonstrates that ENTPD5 knockdown can suppress the proliferation and migration ability of SOC by inhibit GRP78/p-eIF-2α/CHOP pathway (Fig. 6C). Based on the results of this study, we recommend further research on the relationship between ENTPD5 and ovarian cancer intercell environment homeostasis, which may be a new promising treatment for human SOC.

## Supplementary Information

**Additional file 1: Table S1.** Distribution of tumor characteristics for ovarian cancer patients. **Table S2.** Primer sequences for RT-qPCR.

## Data Availability

Not applicable.

## Abbreviations

ENTPD5: Ectonucleoside Triphosphate Diphosphohydrolase 5
OC: Ovarian cancer
SOC: Serous ovarian cancer
FT: Normal fallopian tube tissues
IHC: Immunohisto-chemistry
qRT-PCR: Quantitative real-time PCR
NC: Negative control
MMP: Matrix metalloproteinase
UPR: Unfolded protein response
ERS: Endoplasmic reticulum stress
GRP78: Glucose-regulated protein 78
CHOP: C/EBP-homologous protein
eIF-2α: Eukaryotic translation initiation factor 2 alpha
