# Supplementary material for: Knockdown of ENTPD5 inhibits tumor metastasis and growth via regulating the GRP78/p-eIF-2α/CHOP pathway in serous ovarian cancer
Source: J Ovarian Res. 2022 Jun 7;15:69. doi: 10.1186/s13048-022-00996-0 (PMC9171961; doi:10.1186/s13048-022-00996-0)
Supplement: Supplementary file 1 — Additional file 1: Table S1. Distribution of tumor characteristics for ovarian cancer patients. Table S2. Primer sequences for RT-qPCR. [file 13048_2022_996_MOESM1_ESM.docx]

| Table S1 Distribution of tumor characteristics for ovarian cancer patients | | |
| --- | --- | --- |
| Variable | Number of patients | |
|  | n | % |
| Histological type | | |
| Serous cystadenocarcinoma | | 79.27 |
| low-grade serous carcinoma | 0 |  |
| high-grade serous carcinoma | 65 |  |
| Mucinous cystadenocarcinoma | 10 | 12.20 |
| Endometrioid tumor | 3 | 3.66 |
| Clear cell carcinoma | 4 | 4.88 |
| FIGO stage | | |
| Stage Ⅰ | 17 | 20.73 |
| Stage Ⅱ | 9 | 10.98 |
| Stage Ⅲ | 49 | 59.76 |
| Stage Ⅳ | 7 | 8.54 |
| age(years) | | |
| <56 | 42 | 51.22 |
| ≥56 | 40 | 48.78 |
| CA125(U/ml) | | |
| <600 | 53 | 64.63 |
| ≥600 | 29 | 35.37 |
| Lymph node metastasis | | |
| Negative | 63 | 76.83 |
| Positive | 19 | 23.17 |
| Omentum metastasis | | |
| Negative | 33 | 40.24 |
| Positive | 49 | 59.76 |

| Table S2 Primer sequences for RT-qPCR | | |
| --- | --- | --- |
| Gene | Forward(5′-to-3′) | Reverse(5′-to-3′) |
| 18s | AGTCCCTGCCCTTTGTACACA | CGATCCGAGGGCCTCACTA |
| ENTPD5 | TGATTCTGTGAAGCCAGGAC | ATTGAGTCTTTGGCCACCTC |
| MMP2 | TACAGGATCATTGGCTACACACC | GGTCACATCGCTCCAGACT |
| MMP7 | GAGTGAGCTACAGTGGGAACA | CTATGACGCGGGAGTTTAACAT |
| MMP9 | TGTACCGCTATGGTTACACTCG | TGTACCGCTATGGTTACACTCG |
